# *MiR-150-5p* attenuates heart failure by targeting MMP14 to regulate vascular smooth muscle cell function

**DOI:** 10.1007/s11010-025-05417-6

**Published:** 2025-10-21

**Authors:** Xu Yu, Yang Zhao, Qikui Wang, Xin Jiang, Liang Zhang

**Affiliations:** Department of Cardiothoracic and Vascular Surgery, Anhui Provincial Chest Hospital, No. 397 Jixi Road, Shushan District, Hefei, 230031 Anhui China

**Keywords:** Heart failure, *miR-150-5p*, MMP14, TAC, HASMCs

## Abstract

*MiR-150-5p* is a microRNA that plays an important role in the heart diseases. However, its specific role and molecular mechanism in heart failure (HF) remain unclear. In this study, we found that *miR-150-5p* was downregulated in patients with HF, while the expression of MMP14 was elevated. In vitro experiments have shown that *miR-150-5p* directly targets MMP14 and inhibits its expression in human aortic smooth muscle cells (HASMCs). Functionally, *miR-150-5p* promotes the proliferation, migration and apoptosis of HASMC, which is a key process in the progression of HF. In the HF mouse model induced by transverse aortic contraction (TAC), overexpression of *miR-150-5p* can improve cardiac function, reduce hypertrophy and fibrosis, while its inhibition aggravates these effects. These findings determined that *miR-150-5p* is a protective regulator in HF, functioning by inhibiting MMP14, and indicated its potential as a therapeutic target for HF treatment.

## Introduction

Heart failure (HF) is the leading cause of hospitalization worldwide, with 3.6 million newly diagnosed patients each year, imposing a socio-economic burden of billions of euros annually [1]. HF is a complex clinical syndrome characterized by the heart’s inability to pump sufficient blood to meet the body’s metabolic demands or to do so only at elevated filling pressures [2]. It results from structural or functional impairments in ventricular filling or ejection, often due to conditions such as coronary artery disease, hypertension, or cardiomyopathies [3]. Pressure overload-induced cardiac remodeling refers to the structural and functional adaptations of the heart in response to chronic mechanical stress, typically caused by conditions such as hypertension, aortic stenosis, or pulmonary hypertension [4]. When the myocardium is subjected to sustained pressure overload, the left ventricle (LV) undergoes concentric hypertrophy, characterized by thickening of the ventricular wall without significant chamber dilation. This compensatory response initially maintains cardiac output by normalizing wall stress (Laplace’s law) but may progress to maladaptive remodeling, leading to diastolic dysfunction, fibrosis, and eventual heart failure—particularly heart failure with preserved ejection fraction (HFpEF) [5].

MicroRNAs (miRNAs) are a class of small non-coding RNAs discovered in recent years, and their evolutionary processes occur in closely related species [6]. Studies have shown that it is involved in various cellular biological processes, such as development, cycle regulation, proliferation, differentiation, and regulation [7]. Recent studies suggest that *miR-150-5p* may play a key role in myocardial remodeling [8]. Studies have found that there is a strong negative correlation between the intensity of left ventricular remodeling and the concentration of plasma *miR-150-5p* [9]. Restoring *miR-150-5p* in mice with myocardial infarction can improve cardiac function of mice, and can alleviate the pathological changes of the myocardium and reduce the apoptosis of myocardial cells [10]. *MiR-150-5p* can exert cardiac protective functions by directly inhibiting the pro-apoptotic genes *Egr2* (ischemia-induced zinc-binding transcription factor) and *P2* × *7r* (pro-inflammatory adenosine triphosphate receptor) in cardiomyocytes [11]. *MiR-150-5p* was first demonstrated in hepatoma cells to inhibit the infiltration and metastasis of tumor cells by influencing the activity of MMP-14 [12]. Another study showed that down-regulating the expression of miR-150-5p could increase the activity of MMP-14 and promote the metastasis and progression of lung squamous cell carcinoma [13]. However, there are no evidence to demonstrate whether *miR-150-5p* is involved in regulating the activity of MMP-14 and its specific mechanism in cardiac tissue.

In this study, we first detected the expressions of *miR-150-5p* and MMP14 in patients with HF and determined that the expression of *miR-150-5p* was negatively correlated with HF, while the expression of MMP14 was positively correlated with HF. Overexpression of *miR-150-5p* significantly reduces the expression of MMP14 and promotes the proliferation, migration and apoptosis of HASMCs. Echocardiography and staining of heart in mice showed that *miR-150-5p* significantly increased cardiac function and inhibited cardiac hypertrophy. These results provide new evidence for *miR-150-5p* as a clinical biomarker and a molecular therapeutic target of HF.

## Methods

### Cell culture and transfection

Human aortic smooth muscle cells (HASMCs) (ATCC, American Type Culture Collection) were cultured in DMEM containing 10% fetal bovine serum, 100 U/ml penicillin, and 100 µg/ml streptomycin. The cells were incubated at 37 °C in a humidified atmosphere of 95% O_2_ and 5% CO_2_. HASMCs were cultured into six-well plates, transfected with miRNAs using Lipofectamine 2000 according to the manufacturer’s instructions.

## RNA extraction and quantitative real-time PCR

After transfection, cells were cultured for 48 h, washed three times with pre-cooled PBS, and lysed in Trizol reagent (Invitrogen, USA) for 5 min. Total RNA was isolated by adding chloroform (1:5 v/v), followed by vigorous vortexing for 1 min and incubation at room temperature for 5 min. The samples were then centrifuged at 12,000 × g for 15 min at 4 °C. The aqueous phase was collected and mixed with isopropyl alcohol (1:2 v/v), incubated at − 20 °C for 30 min, and centrifuged again at 12,000 × g for 15 min at 4 °C. The resulting RNA pellet was washed sequentially with anhydrous ethanol and 70% ethanol, air-dried, and dissolved in DEPC-treated water. First-strand cDNA synthesis was performed using the First Strand cDNA Synthesis Kit (Cat# 11141ES10, YEASEN, China) following the manufacturer’s protocol. Quantitative real-time PCR (qRT-PCR) was carried out using SYBR Green Master Mix (Low Rox Plus) (Cat# MQ10201S, Monad, China) on a QuantStudio® 12 K Flex Real-Time PCR System (Thermo Fisher Scientific, USA). All reactions were conducted in triplicate, and gene expression levels were normalized to GAPDH.

## Protein extraction and western blot analysis

Cells were washed three times with ice-cold PBS and lysed in RIPA buffer (25 mM Tris–HCl [pH 8.0], 150 mM NaCl, 1% Triton X-100, 0.2% sodium deoxycholate, 1 mM DTT, 5 mM EDTA, 0.5 mM PMSF, 10 mM NEM, 10 mM iodoacetamide, and protease inhibitor cocktail) for 20 min on ice. Lysates were centrifuged at 12,000 g for 10 min at 4 °C. Protein samples were mixed with 1 × SDS-loading buffer, boiled for 15 min, and analyzed with SDS-PAGE electrophoresis and western blotting.

## Enzyme-linked immunosorbent assay (ELISA)

The patient’s blood samples were centrifuged at 2,000 rpm for 10 min at 4 °C, and the supernatant was collected to detect the secretion of MMP14 in the serum using the human MMP14 detection kit.

## Animal model

The TAC model of myocardial remodeling was established using 8-week-old mice. Before the operation, the mice were anesthetized with an intraperitoneal injection of sodium pentobarbital (50 mg/kg body weight). A small incision was made in the thoracic cavity of the mouse to expose the aortic arch, and at the same time, a small animal ventilator (ALC-V8S, ALCBIO, China) was used to maintain the breathing of the mouse. TAC is performed by covering the arch of the foot with a 7–0 thread suture attached to a 27-point needle between the brachial trunk and the left common carotid artery. Afterwards, the muscles and skin were sutured carefully in sequence to wake the mice up and use them for subsequent experiments. Mice that died within the first day after the operation were excluded from further studies. In the TAC model of miRNA treatment, miR-150-5p was delivered via tail vein injection of rAAV9-miR-150-5p (1 × 10^11^ vg/mouse) 1 week pre-TAC. Seven days after the operation, the mice were examined by echocardiography and sacrificed. Euthanasia was performed by anesthesia with injection of 3% chloral hydrate (0.1 ml/10 g body weight) followed by cervical dislocation. Animal experiments conformed to the guidelines of the Care and Use of Animals for Research by the Ministry of Science and Technology of the P. R. China (2006–398) and Reporting In Vivo Experiments (ARRIVE) guidelines. Animal care and experimental procedures were approved by Anhui Provincial Chest Hospital (Approve number: KJ2024-129).

## Echocardiogram

Echocardiography was performed by a researcher who was unaware of the treatment using the CMS1700C high-resolution micro-ultrasound system (ESAOTE, Italy). While mice were anesthetized with an intraperitoneal injection of sodium pentobarbital (50 mg/kg body weight). After the mice were anesthetized, the chest was treated with a depilatory agent, and images of the left ventricle were obtained on the long axis using the sensor of CMS1700C.

## Pathological staining methods

The hearts of mice were fixed overnight with 4% paraformaldehyde, embedded in paraffin, and sectioned (3 mm). The cross-sections of the heart were stained with hematoxylin, eosin (H&E), and Sirius red.

## Statistical analysis

GraphPad Prism 8 was used for statistical analysis. The data are shown as mean ± SD. The comparison between two groups were made by unpaired two-tailed *t*-tests. To compare the means of more than two groups, we used one-way ANOVA tests with Bonferroni adjustment for multiple testing. *P* < 0.05 was considered significant.

## Results

### The expression of *miR-150-5p* was downregulated and the expression of MMP14 was upregulated in patients with HF

**Fig. 1:**
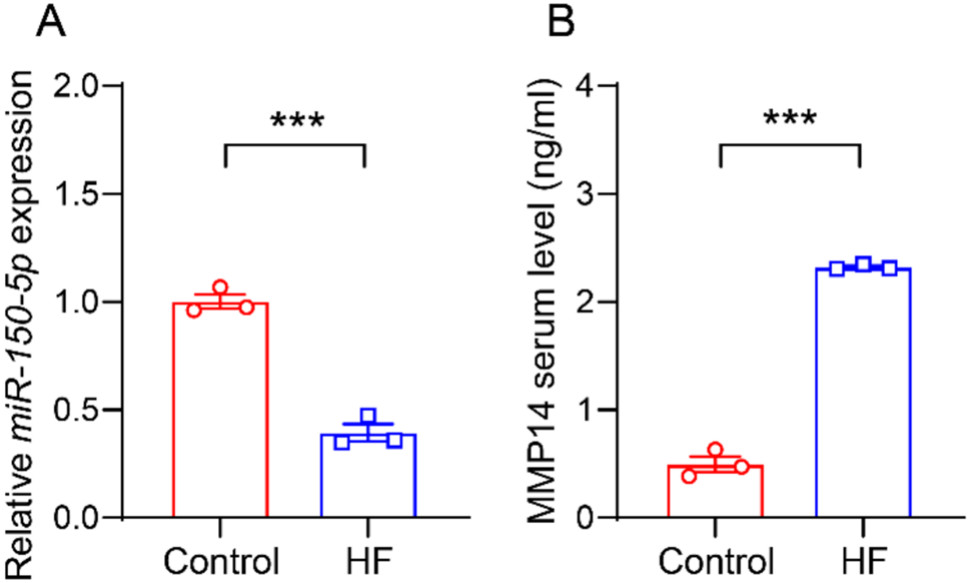
*miR-150-5p* was downregulated and MMP14 was upregulated in patients with heart failure (HF). **A** RT-qPCR analysis to examine the expression of *miR-150-5p* in patients with or without HF. n = 3. **B** ELISA to measure secreted serum MMP14 of patients with or without HF. n = 3. ***P < 0.001. unpaired two-tailed *t*-test

To explore the relationship between *miR-150-5p* and heart failure, we collected cardiac tissues from clinical patients with heart failure for analysis. RT-qPCR analysis showed that compared with normal controls, the expression of *miR-150-5p* in HF patients was significantly downregulated (Fig. 1A). Previous studies have shown that *miR-150-5p* can regulate the expression of MMP14 in liver cancer [12], and its important role in myocardial infarction [14, 15]. We suspect that *miR-150-5p* may be involved in the occurrence and development of HF by regulating MMP14. To verify this conjecture, we detected the expression of MMP14 in patients with heart failure. ELISA analysis showed that the level of MMP14 in the serum of HF patients was significantly higher than that of the normal controls (Fig. 1B). These results indicate that the expressions of *miR-150-5p* and MMP14 are significantly associated with HF.

## *miR-150-5p* inhibits the expression of MMP14

**Fig. 2:**
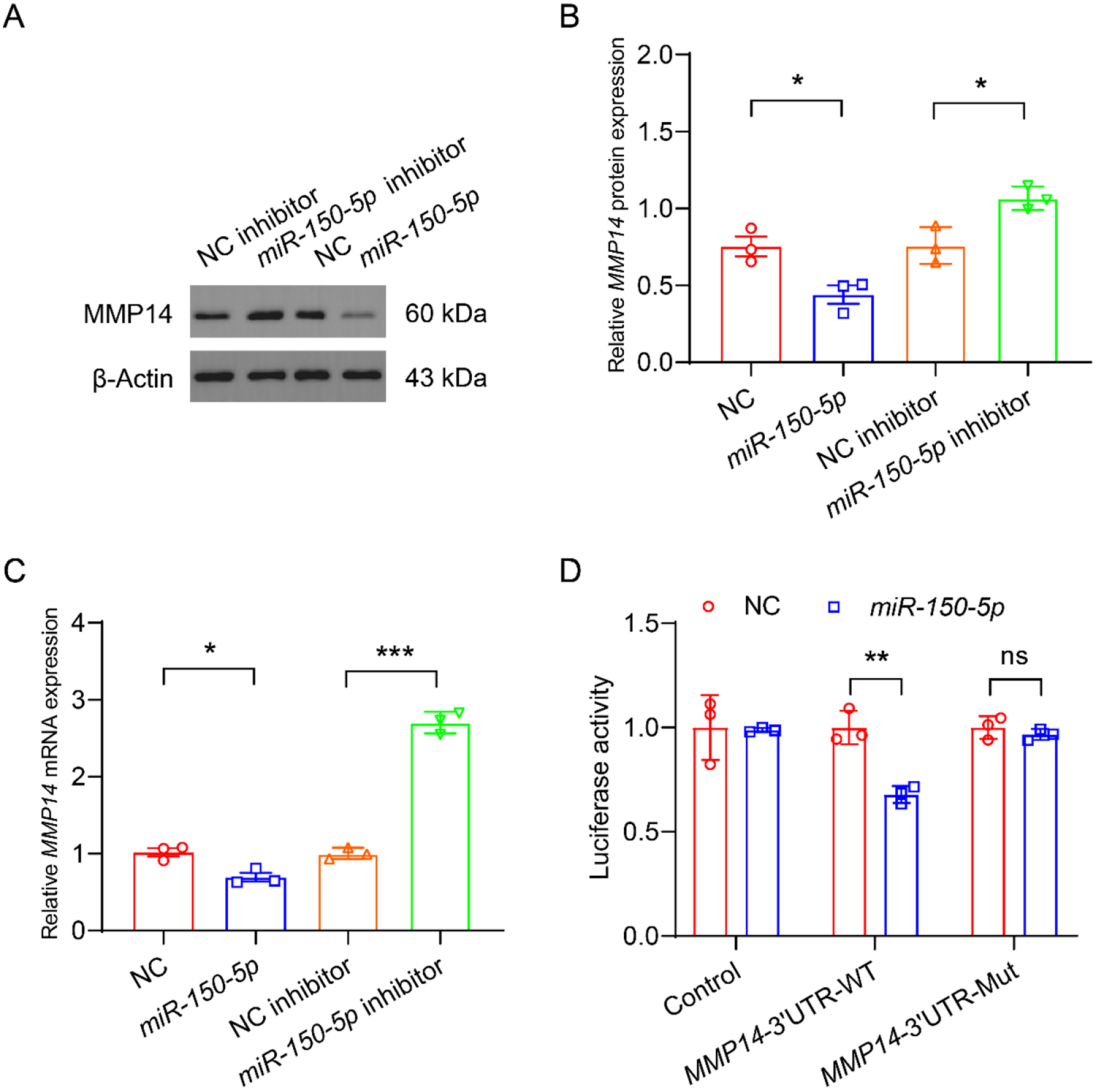
*miR-150-5p* promotes the expression of MMP14. **A** Western blot analysis to examine the protein expression of MMP14 of HASMCs transfected with NC, *miR-150-5p*, NC inhibitor, and *miR-150-5p* inhibitor. **B** Quantification of (**A**). n = 3. **C** RT-qPCR analysis to examine the mRNA expression of *MMP14* of HASMCs transfected with NC, *miR-150-5p*, NC inhibitor, and *miR-150-5p* inhibitor. **D** Luciferase activity of *MMP14*−3’UTR-WT or *MMP14*−3’UTR-Mut reporters in the presence of *miR-150-5p* vs. NC. n = 3.*P < 0.05, **P < 0.01, ***P < 0.001. One-way ANOVA

To explore whether *miR-150-5p* regulates the expression of MMP14, we transfected *miR-150-5p* and *miR-150-5p* inhibitor into human aortic smooth muscle cells (HASMCs) and detected the expression of MMP14. Western blot analysis showed that compared with the NC group, *miR-150-5p* significantly decreased the protein expression level of MMP14 (Fig. 2A-B). Compared with the NC inhibitor group, *miR-150-5p* inhibitor significantly increased the protein expression level of MMP14 (Fig. 2A-B). RT-qPCR analysis showed that compared with the NC group, *miR-150-5p* significantly decreased the mRNA expression level of *MMP14* (Fig. 2C). Compared with the NC inhibitor group, *miR-150-5p* inhibitor significantly increased the mRNA expression level of *MMP14* (Fig. 2C). These results indicate that *miR-150-5p* inhibits the expression of MMP14. Moreover, dual-luciferase assay showed that *miR-150-5p* significantly reduced the activity of MMP14-3’UTR-WT, but did not affect the activity of MMP14-3’UTR-Mut (Fig. 2D). This result indicates that *miR-150-5p* regulates the expression of MMP14 by binding to its 3’UTR region.

## *miR-150-5p* promotes the proliferation and apoptosis of HASMCs

**Fig. 3:**
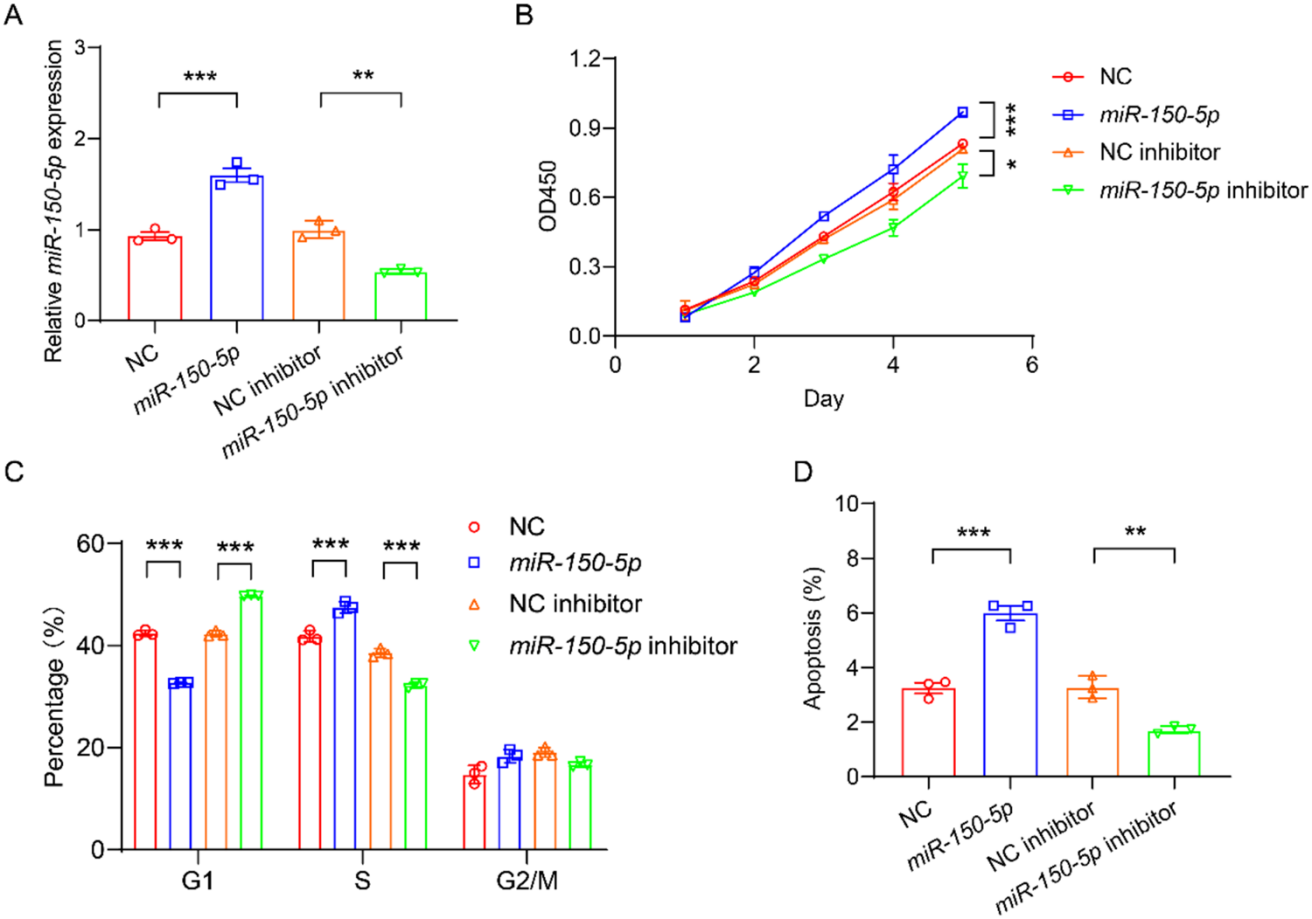
*miR-150-5p* promotes the proliferation of smooth muscle cells and inhibits apoptosis. **A** RT-qPCR analysis to examine the expression of *miR-150-5p* in HASMCs transfected with NC, *miR-150-5p*, NC inhibitor, and *miR-150-5p* inhibitor. n = 3. **B** CCK8 assay to examine the proliferation of HASMCs transfected with NC, *miR-150-5p*, NC inhibitor, and *miR-150-5p* inhibitor. n = 3. **C** Flow cytometry to examine the cell cycle of HASMCs transfected with NC, *miR-150-5p*, NC inhibitor, and *miR-150-5p* inhibitor. n = 3. **D** Flow cytometry to examine the apoptosis of HASMCs transfected with NC, *miR-150-5p*, NC inhibitor, and *miR-150-5p* inhibitor. n = 3. *P < 0.05, **P < 0.01, ***P < 0.001. One-way ANOVA

To explore how *miR-150-5p* leads to HF, we examined the cellular functions of HASMCs. RT-qPCR analysis showed that *miR-150-5p* mimics significantly increased the expression of *miR-150-5p*, while *miR-150-5p* inhibitor significantly decreased the expression of *miR-150-5p* (Fig. 3A). CCK8 assay showed that *miR-150-5p* significantly increased the cell number of HASMCs, and the *miR-150-5p* inhibitor significantly decreased the cell number of HASMCs, indicating that *miR-150-5p* promoted the proliferation of HASMCs (Fig. 3B). To verify this result again, we examined the effect of *miR-150-5p* on the cell cycle of HASMCs. Flow cytometry analysis showed that compared with the NC group, *miR-150-5p* significantly reduced the number of cells in the G1 phase and increased the number of cells in the S phase (Fig. 3C). Compared with the NC inhibitor group, the *miR-150-5p* inhibitor significantly increased the number of cells in the G1 phase and decreased the number of cells in the S phase (Fig. 3C). These results once again confirm that *miR-150-5p* can promote the proliferation of HASMCs. Flow cytometry analysis showed that *miR-150-5p* significantly increased the number of apoptotic HASMCs cells, while *miR-150-5p* inhibitor significantly reduced the number of apoptotic HASMCs cells, indicating that *miR-150-5p* promoted the apoptosis of HASMCs (Fig. 3D).

## *miR-150-5p* promotes the migration of HASMCs

**Fig. 4:**
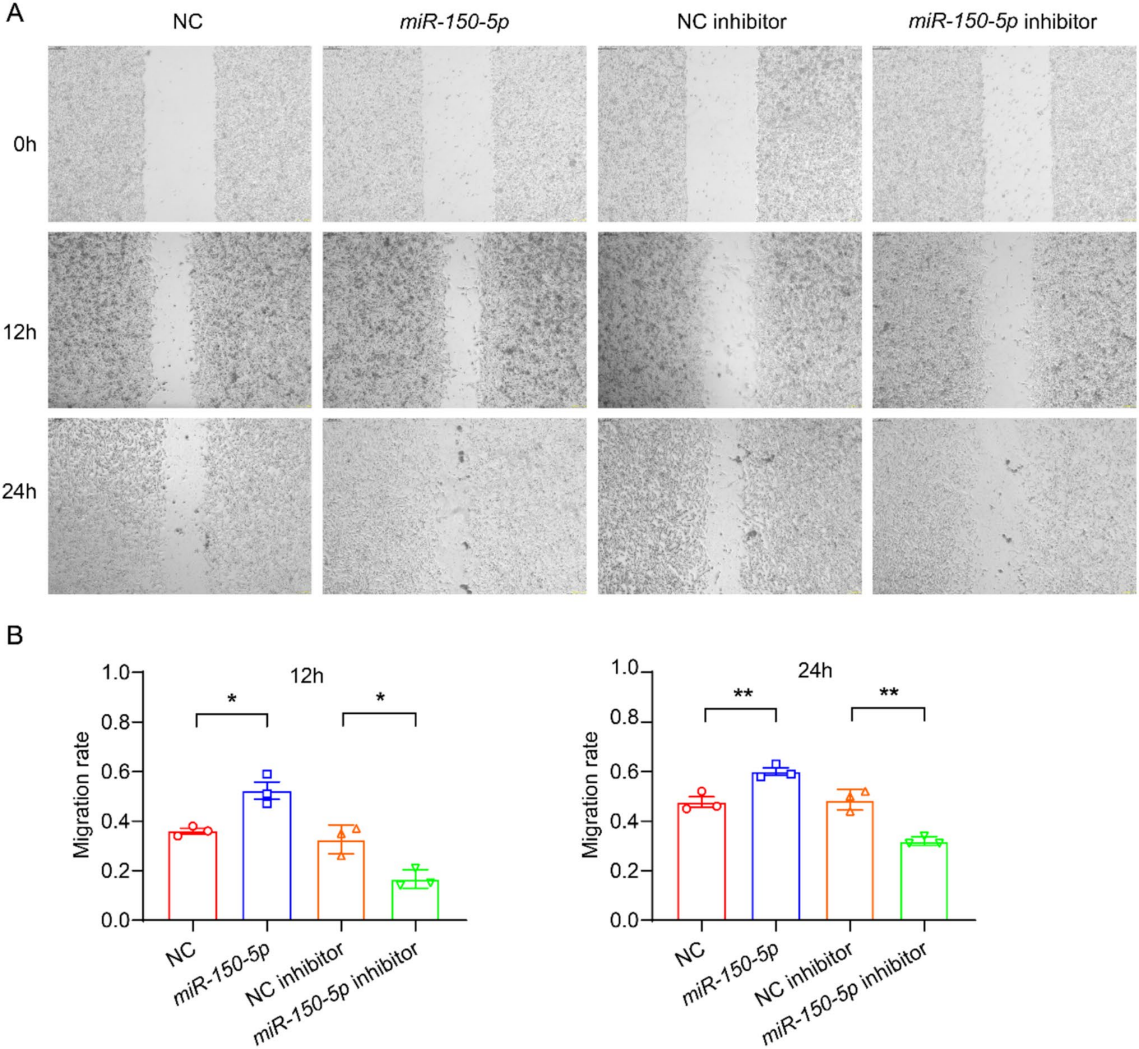
*miR-150-5p* promotes the migration of smooth muscle cells.** A** Raw images of HASMCs transfected with NC, *miR-150-5p*, NC inhibitor, and *miR-150-5p* inhibitor at the 0-, 12-, and 24-h time points during their migration. **B** The area of the changes in the healed wound shown in (**A**) was quantified and plotted. n = 3. *P < 0.05, **P < 0.01. One-way ANOVA

To explore the effect of *miR-150-5p* on the migration ability of HASMCs, we conducted a cell scratch assay. The results showed that at both 12 h and 24 h, *miR-150-5p* significantly increased the migration ability of HASMCs, while *miR-150-5p* inhibitor significantly decreased the migration ability of HASMCs (Fig. 4A-B). Based on the above results, we provide evidence to prove that *miR-150-5p* is involved in the occurrence of HF by regulating the cellular functions of HASMCs, including promoting proliferation, apoptosis, and migration, which regarded as factors that accelerates HF.

## *miR-150-5p* improves the cardiac function of mice

**Fig. 5:**
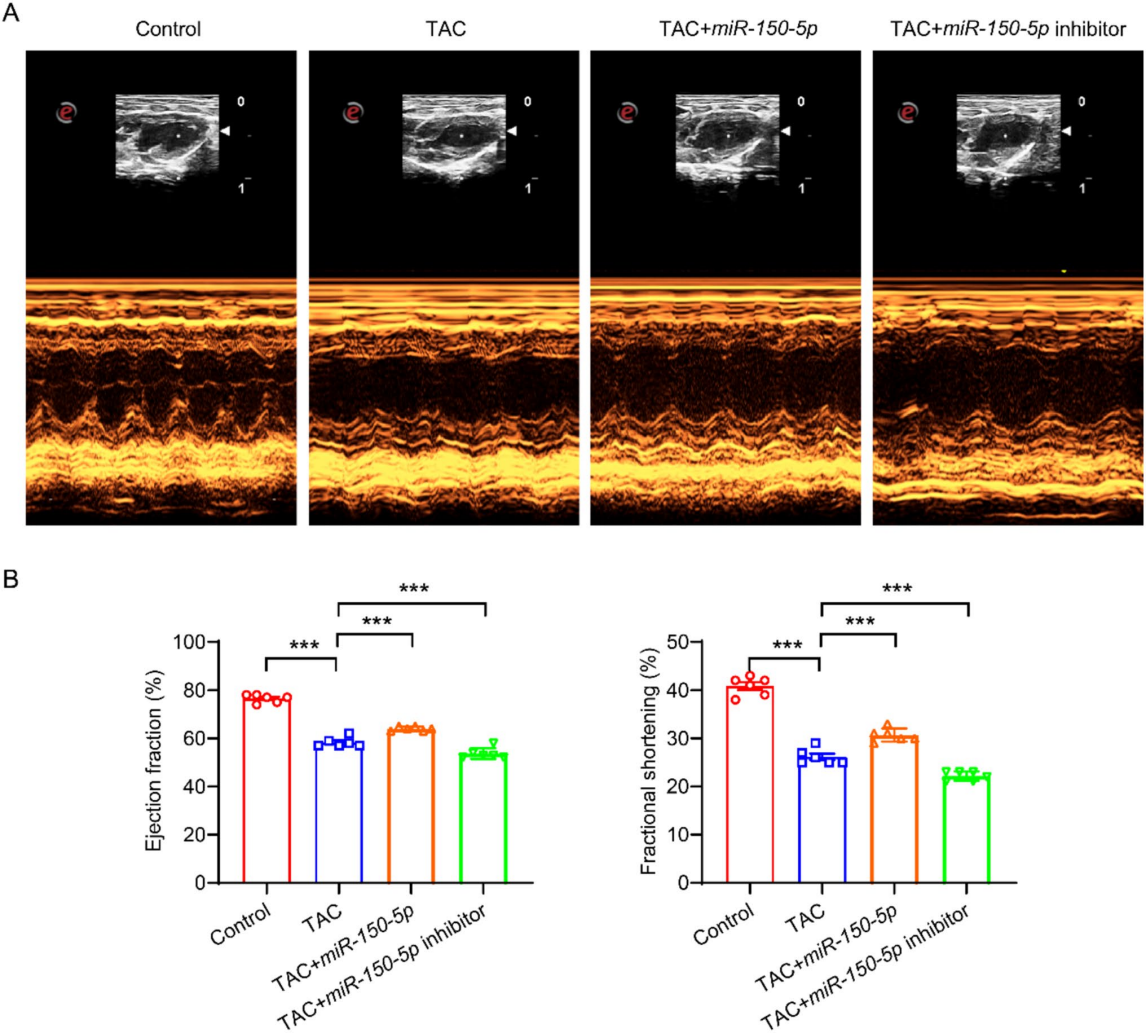
*miR-150-5p* promotes cardiac function. **A** Echocardiography of the heart of mice treated with control, TAC, TAC + *miR-150-5p*, and TAC + *miR-150-5p* inhibitor. **B** LVEF and LVFS of mice treated with control, TAC, TAC + *miR-150-5p*, and TAC + *miR-150-5p* inhibitor. n = 6. ***P < 0.001. One-way ANOVA

To demonstrate the effect of *miR-150-5p* on HF in vivo, we induced HF in mice using the TAC (Transverse Aortic Constriction) model. TAC is a commonly used experimental model for mice or rats, which is used to simulate HF and myocardial hypertrophy caused by pressure overload [16]. Echocardiography showed that left ventricular ejection fraction (LVEF) and left ventricular fraction shortening (LVFS) of mice in the TAC model group decreased significantly (Fig. 5A-B), indicating the successful construction of the model. Compared with the TAC model group, *miR-150-5p* significantly increased the LVEF and LVFS, while *miR-150-5p* inhibitor significantly decreased the LVEF and LVFS. These results indicate that *miR-150-5p* promotes cardiac function in vivo.

## *miR-150-5p* inhibits cardiac hypertrophy

**Fig. 6:**
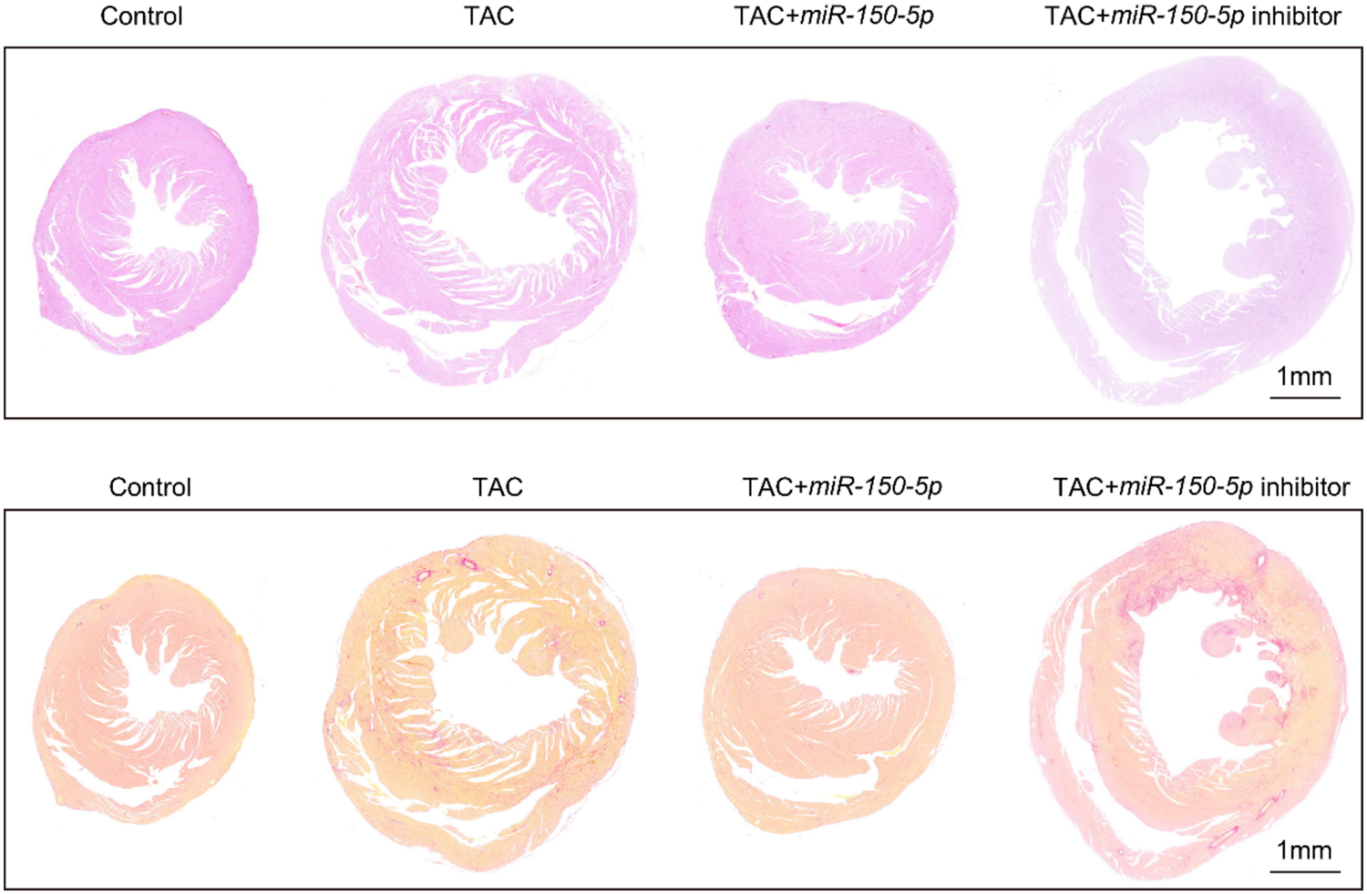
*miR-150-5p* inhibits cardiac hypertrophy. **A** H&E staining of the heart of mice treated with control, TAC, TAC + *miR-150-5p*, and TAC + *miR-150-5p* inhibitor. **B** Sirius red staining of mice treated with control, TAC, TAC + *miR-150-5p*, and TAC + *miR-150-5p* inhibitor

H&E staining showed that compared with WT mice, TAC modeled mice showed more severe cardiac hypertrophy (Fig. 6A). Compared with the TAC model group, *miR-150-5p* significantly inhibited cardiac hypertrophy, while *miR-150-5p* inhibitor significantly promoted cardiac hypertrophy (Fig. 6A). Sirius red staining showed that compared with WT mice, TAC modeled mice showed more extensive myocardial fibrosis (Fig. 6B). Compared with the TAC model group, *miR-150-5p* significantly inhibited myocardial fibrosis, while *miR-150-5p* inhibitor significantly promoted myocardial fibrosis (Fig. 6B). These results indicate that *miR-150-5p* inhibits cardiac hypertrophy and fibrosis in vivo.

## Discussion

This study revealed the key role of the *miR-150-5p*/MMP14 regulatory axis in the pathogenesis of HF through clinical and mechanism research. We demonstrated that *miR-150-5p* was significantly downregulated in the cardiac tissues of HF patients, while MMP14 was upregulated (Fig. 1), thereby establishing their clinical correlation. Our findings indicate that *miR-150-5p* directly targets MMP14 through 3’UTR binding and jointly accelerates the progression of HF by regulating the function of human aortic smooth muscle cells (HASMCs), including promoting proliferation, migration and apoptosis (Figs. 2, 3, 4). Through the tac induced HF mouse model, we provided evidence that the overexpression of miR-150-5p could not only rescue cardiac function by improving LVEF and LVFS, but also significantly alleviate cardiac hypertrophy and fibrosis (Figs. 5, 6). These findings fundamentally advance our understanding of miRNA-mediated HF progression by revealing the new *miR-150-5p*/MMP14 pathway, while emphasizing the dual potential of *miR-150-5p* as a diagnostic biomarker and a promising therapeutic target for HF regulation through vascular smooth muscle.

*MiR-150-5p* shows unique advantages as a therapeutic target for heart failure, the core of which lies in its pleiotropic regulatory mechanism. Unlike single-target drugs, *miR-150-5p* can intervene in the pathological process of heart failure more comprehensively by simultaneously regulating MMP14 and its downstream networks (which may involve pathways such as ECM remodeling, inflammatory response, and apoptosis). The synergistic improvement effect we observed in the study on myocardial function, fibrosis and hypertrophy suggests that it may overcome the problem of target limitations in the current treatment of heart failure. However, this strategy also faces significant challenges: The first is the issue of delivery efficiency, how to achieve the specific enrichment of miRNA in cardiac tissues (especially in fibrotic regions); Secondly, there is the accuracy of dose control. Considering the “proliferation-apoptosis biphasic regulation” exhibited by *miR-150-5p* on HASMCs, its therapeutic window may be relatively narrow. The newly developed exosome carriers or heart-targeted lipid nanoparticles may offer solutions to these problems, but further pharmacokinetic studies are needed for verification.

From the perspective of translational medicine, the therapeutic development of *miR-150-5p* needs to address three key issues: timing selection, patient stratification, and combination medication. Our data show that *miR-150-5p* has a significant effect in the early intervention of the TAC model, suggesting that it may be more suitable for the preventive treatment of stage B heart failure (the asymptomatic period of structural heart disease). Patient stratification based on the expression level of MMP14 may improve the treatment accuracy because there are significant individual differences in the *miR-150-5p*/MMP14 axis revealed by this study. It is worth noting that *miR-150-5p* may have a synergistic effect with existing heart failure drugs (such as ARNI or SGLT2 inhibitors): the former targets cell remodeling, and the latter improves metabolic abnormalities [17]. This multi-target combined strategy represents the direction of the next generation of heart failure treatment. However, it is necessary to be vigilant that the tumor suppressor effect of *miR-150-5p* in tumors such as liver cancer suggests that its long-term safety needs to be strictly evaluated, especially the potential impact on the high-risk population of cancer requires the establishment of a complete monitoring system. However, our study has limitations, in the in vitro experiments, only HASMCs were used to verify the regulatory effect of miR-150-5p on MMP14, and core cardiac functional cells such as cardiac fibroblasts and cardiomyocytes were not involved. Due to significant differences in biological characteristics and regulatory networks between cells from different tissue sources, the conclusions obtained from HASMCs cannot be directly extended to cardiac tissue, which may lead to an incomplete understanding of the mechanism of action of miR-150-5p in HF (especially myocardial fibrosis). Future studies need to further use primary cardiac fibroblasts, cardiomyocytes, or construct heart-specific cell models to deeply explore the regulatory effect of *miR-150-5p* on the functions of these core cells, clarify its specific molecular mechanisms in myocardial fibrosis and myocardial hypertrophy, and thus more comprehensively elucidate the regulatory network of *miR-150-5p* in HF.

Overall, we have provided strong evidence of *miR-150-5p* and MMP14 in clinical practice related to HF, and identified the *miR-150-5p*/MMP14 axis as the molecular mechanism by which it regulates HF. Functionally, the *miR-150-5p*/MMP14 axis participates in the occurrence and development of HF by regulating the proliferation, apoptosis and migration of HASMCs. These results provide new targets and strategies for the clinical treatment of HF.

## Data Availability

Any additional information required to reanalyze the data reported in this paper is available from the corresponding author upon request.
